# Diabetics without prior cardiovascular disease have diffuse interstitial fibrosis by CMR independent of clinical risk factors

**DOI:** 10.1186/1532-429X-14-S1-O84

**Published:** 2012-02-01

**Authors:** Ravi Shah, Ajay Rao, Michael Jerosch-Herold, Raymond Kwong, Gail K Adler

**Affiliations:** 1 Brigham and Women's Hospital, Boston, MA, USA

## Background

As a precursor to overt heart failure, diabetics experience a unique alteration in myocardial architecture marked by diffuse interstitial fibrosis. We hypothesized that the myocardial extracellular volume fraction (MECVF) could be used to detect myocardial matrix remodeling and its relationship to ventricular structure and function in diabetics without cardiovascular disease.

## Methods

Diabetics without any history of MI (by clinical grounds or by evidence of subendocardial LGE) or heart failure underwent 3T CMR imaging and assessment of MECVF and assessment of biochemical parameters of insulin sensitivity. 10 volunteers without diabetes, hypertension, or clinical cardiac disease were used for comparison. Cine SSFP, LGE imaging, and myocardial T1 quantitation using a modified Look-Locker sequence were performed. For measurement of MECVF, segmental myocardial T1 was measured once pre-Gd and at least 3 times over 3-20 minutes post-Gd (gadopentetate dimeglumine, 0.15 mmol/kg). Regression of myocardial R1 (=1/T1) on R1 in the blood pool was used to determine the Gd partition coefficient, which when multiplied by (1-hematocrit/100), gave MECVF. Left atrial volume index (LAVI) and emptying function, as well as LV and RV volumes and function, were determined by standard techniques.

## Results

55 recruited patients were predominantly male (62%), with an average age of 54±8 years, systolic blood pressure 126±14 mmHg, body mass index (BMI) 32.4±4 kg/m2, fasting glucose 108±25 mg/dl, and triglycerides 128±69 mg/dl. CMR LVEF averaged 60±5%, LV mass index 45±11 g/m2, LVEDV index 68±14 ml/m2, LAVI 35±9 ml/ m2 (assessed in 38 patients; all values in normal range). MECVF in diabetics was 0.36±0.05 (as compared to normal controls 0.29±0.04, P = .0004 by unpaired t-test). MECVF correlated with LAVI (r =0.34; p = 0.04), but not with LV mass index, fasting glucose or insulin levels, insulin sensitivity, triglycerides, LVEF, age, BMI, LA function or systolic blood pressure.

## Conclusions

Well-controlled diabetics with normal ventricular structure, function, and no prior cardiac disease have diffuse interstitial myocardial fibrosis by CMR. Diffuse fibrosis is associated with LAVI, a marker of diastolic function, but not with other markers of clinical or cardio-metabolic risk. Diffuse myocardial damage occurs early in the course of diabetic cardiomyopathy independent of hypertension and obesity, and may be a potential target for early intervention in at-risk diabetic patients with subclinical myocardial disease.

## Funding

RVS was supported by a Post-Doctoral Fellowship Grant from the American Heart Association (11POST000002). RYK, MJ, GKA are supported by grants from the National Institutes of Health.

